# Supplementary material for: Effectiveness of Brodalumab for the Treatment of Moderate-to-Severe Psoriasis: A Retrospective, Real-World Multicenter Study with a Focus on Obese and Multi-Failure Patients—IL PSO (Italian Landscape Psoriasis)
Source: J Clin Med. 2025 Feb 8;14(4):1087. doi: 10.3390/jcm14041087 (PMC11856090; doi:10.3390/jcm14041087)
Supplement: Supplementary file 1 [file jcm-14-01087-s001.zip › jcm-3424108-supplementary.pdf]

**Adverse Events**	**N (%)**	**Therapy discontinuation**
Tot Patients	15 (5.0%)	
Tot AEs	17	
Latent tubercolosis	4 (23.5%)	1 (25%)
Conjunctuvitis	2 (11.8%)	
ANA 320	1 (5.9%)	
candidiasis of the oral cavity	1 (5.9%)	
nonspecific colitis	1 (5.9%)	1 (100%)
edema of the lower limbs*	1 (5.9%)	1 (100%)
Hypotension*	1 (5.9%)	1 (100%)
maculo papular rash*	1 (5.9%)	1 (100%)
epilepsy	1 (5.9%)	
ererythema	1 (5.9%)	
fatigue	1 (5.9%)	1 (100%)
leukoplakia	1 (5.9%)	1 (100%)
steatosis	1 (5.9%)	

* occurred at the same patient
